# Evolution of resource cycling in ecosystems and individuals

**DOI:** 10.1186/1471-2148-9-122

**Published:** 2009-06-01

**Authors:** Anton Crombach, Paulien Hogeweg

**Affiliations:** 1 Theoretical Biology and Bioinformatics Group, Utrecht University, Padualaan 18, 3584 CH Utrecht, The Netherlands

## Abstract

**Background:**

Resource cycling is a defining process in the maintenance of the biosphere. Microbial communities, ranging from simple to highly diverse, play a crucial role in this process. Yet the evolutionary adaptation and speciation of micro-organisms have rarely been studied in the context of resource cycling. In this study, our basic questions are how does a community evolve its resource usage and how are resource cycles partitioned?

**Results:**

We design a computational model in which a population of individuals evolves to take up nutrients and excrete waste. The waste of one individual is another's resource. Given a fixed amount of resources, this leads to resource cycles. We find that the shortest cycle dominates the ecological dynamics, and over evolutionary time its length is minimized. Initially a single lineage processes a long cycle of resources, later crossfeeding lineages arise. The evolutionary dynamics that follow are determined by the strength of indirect selection for resource cycling. We study indirect selection by changing the spatial setting and the strength of direct selection. If individuals are fixed at lattice sites or direct selection is low, indirect selection result in lineages that structure their local environment, leading to 'smart' individuals and stable patterns of resource dynamics. The individuals are good at cycling resources themselves and do this with a short cycle. On the other hand, if individuals randomly change position each time step, or direct selection is high, individuals are more prone to crossfeeding: an ecosystem based solution with turbulent resource dynamics, and individuals that are less capable of cycling resources themselves.

**Conclusion:**

In a baseline model of ecosystem evolution we demonstrate different eco-evolutionary trajectories of resource cycling. By varying the strength of indirect selection through the spatial setting and direct selection, the integration of information by the evolutionary process leads to qualitatively different results from individual smartness to cooperative community structures.

## Background

Organisms influence their surroundings by taking up nutrients from the environment and excreting waste products in it. As Earth is a closed system with respect to its chemical components, this leads to resource cycles. Moreover, in doing so organisms may create a specific local environment for their offspring and competitors. From an ecological point of view these are rather basic observations, yet the overall consequences of such feedback between organisms and their abiotic environment on the evolution of a population, community and ecosystem are not well-studied.

One possible outcome of such organism-environment interaction is metabolic crossfeeding. Crossfeeding is an indirect interaction between two or more species, usually microorganisms: it is often observed as the dependence between bacterial strains on each others metabolites. Especially determining the ecological preconditions for such cooperative communities has received much attention. Both experimentally [1-3] and theoretically [4-7] it has been shown that crossfeeding may evolve due to trade-offs in resource uptake and processing, but also simply through the excretion of secondary metabolites.

While crossfeeding and related experimental evolution studies [8-14] have been done mostly in 'artificial' well-mixed environments, the last few years metagenomics has been shedding light on the interplay between microbial communities and their 'natural' local environment [15]. Such whole-ecosystem views show various eco-evolutionary solutions on different spatial and temporal scales to nutrient processing [16-18]: shallow phylogenetic divergence, yet large ecological divergence in the human intestine [19], generalist bacterial lineages performing carbon processing [20], but also temporal and spatial specialization through resource partitioning among *Vibrionaceae *strains in coastal waters [21]. Closely related are analyses in evolutionary functional genomics. A striking example is the finding that many bacterial species contain only parts of the citric acid cycle, suggesting extensive metabolic cooperation among bacterial lineages [22].

In this work we use a computational modeling approach to gain a more general, qualitative insight in the spatial and temporal dynamics and mechanisms of evolving organism-environment interactions. Previous studies have shown the importance of an interplay between ecological and evolutionary processes. It plays a crucial role in the generation and turnover of ecological diversity [23,24]. In addition, the spatial locality of ecological and evolutionary processes has been shown to strongly influence the outcome and dynamics of evolutionary processes [24-26]. Furthermore, evolving interactions via resources has been shown to facilitate niche creation and selection on an ecosystem level. Stable genotypic and phenotypic diversity through resource partitioning was shown by [27], while evolution of ecosystems as an example of multilevel selection was investigated by [28,29]. With respect to evolving interactions among individuals, it has been shown that this may lead to niche creation and ecological diversification [30-32].

Hence we include interlocking ecological and evolutionary processes and a spatial embedding of these processes. As we concentrate on the dual feedback between organisms and their (local) environment, we restrict interactions between individuals to competition for reproduction via nutrients. Furthermore, motivated by metagenomic studies showing the dominant role of microbes in the process of nutrient, or resource, cycling [18,33], resources can be altered according to a simple artificial chemistry that allows for cycling. Schematically speaking, individuals reproduce by taking up a resource, processing it and excreting the resulting resource as waste. An environmental feedback is established, and by eating the environment changes and future feeding opportunities in the neighborhood are affected. Importantly, a frustration arises as direct selection for resource processing can be antagonistic to the indirect selection for cycling resources. Thus we have a simple evolving ecosystem, with the important feature that individuals determine how much fitness they derive from a resource and which waste product, that is new resource, they produce.

Note that in contrast to studies on crossfeeding [5,7], we abstract from reduction/oxidation and energy constraints in order to focus the analysis on the qualitative effects that organism-environment interactions have on the eco-evolutionary outcome. Also, with respect to ecological studies on food webs [34-36], our model leaves out any predatory or parasitic relationships between individuals.

In this model, we study the effect of indirect selection. We do this by varying two parameters: the spatial setting and the strength of direct selection. Firstly, we compare local feedback to a null model that lacks this local feedback due to the mixing of individuals, yet still has cycling on a lattice-wide scale. Secondly, we study the relative balance between direct selection for processing resources against indirect selection for cycling. We show that local feedback enhances indirect selection, as it allows individuals to shape their local surroundings. This results in evolutionary stagnation: resource distributions are more in equilibrium and resource cycling is slower than in the null model. Furthermore, local feedback shows a long-term trend for independent, 'smart' individuals. Individuals are adapted at cycling resources themselves, and do so with shorter cycles than individuals from the null model. As such, especially for relatively high indirect selection (i.e. low direct selection) we find single generalists dominating the population eventually. In contrast, the null model – with only a global cycling of resources – displays more turbulent resource dynamics both qualitatively and quantitatively, and a preferred evolution for cooperating, crossfeeding lineages. By shifting the balance of selection pressures by adjusting direct selection, we find similar changes in evolutionary behavior as for the two spatial settings. In both the local feedback and null model, low direct selection results in 'quiet' resource dynamics and an evolutionary trend for self-sufficient individuals. Also, if direct selection increases, resource dynamics become more turbulent and crossfeeding lineages become a favored behavior.

Thus strong indirect selection for resource cycling, both via local feedback and low direct selection, favors the evolution of self-sufficient individuals, while weak indirect selection, accomplished through only a global feedback and strong direct selection, leads to an ecosystem based resource cycling via crossfeeding lineages.

## Methods

**Figure 1:**
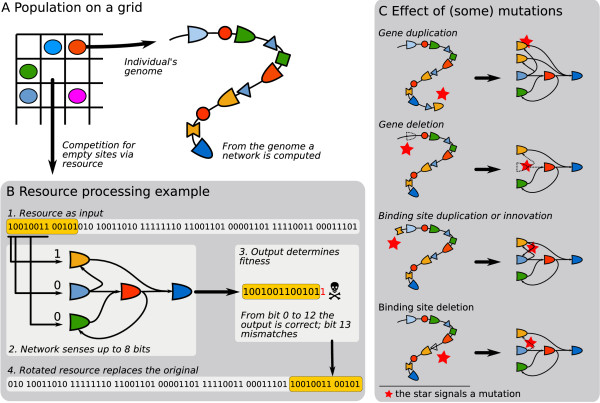
**Overview of the model**. A. Individuals and resources are placed on a grid (size 100×100). Individuals consist of a genome, from which a network is computed. They compete for reproduction into empty grid sites by processing resources. B. The resource is a bit string of length 64. Maximally the first 8 bits can be sensed by a network, which then produces a sequence of bits at its output. The output is matched to the original bit string, and the length of the correct sequence (matching the bit string from the leftmost bit) is the raw score of the individual, which in this example is 13. If the individual reproduces, the resource is rotated from right to left for 13 bits and placed back in the grid site. C. The effect of a few types of mutation on the genome (left) and the topology of the network (right). By default the parameter values for each type of mutation are: gene duplication 16·10^-4^, deletion 24·10^-4^, binding specificity 4·10^-4^, gene expression threshold 4·10^-4^, binding site duplication 4·10^-4^, deletion 10·10^-4^, innovation 1·10^-4^, binding specificity 4·10^-4^, weight 4·10^-4^. In order to balance the growth of the network, we apply a small penalty per gene and binding site of *pen *= 2.5·10^-5^.

We describe our model from a high-level perspective first, followed by several sections covering the details. Central to the local and null model is the processing of resources: organisms have to evolve their regulatory network such that they gain energy from nutrients in the environment. As depicted in Figure 1B, a resource is a bit string, and as an abstraction of metabolic activity an individual has to reproduce the bit string as a temporal output (expression) pattern of its gene regulatory network. We name this a "bite". The example in Figure 1B shows a bite of 13 bits. Next, this bite determines both the fitness of an individual and what waste product is left in the environment: the bite is cut from the left of the resource and re-attached at the right side, effectively rotating the resource bit string.

Thus we combine a model of genes and binding sites on a genome, that are translated into a regulatory network, with a very abstract approach to metabolic processing: processing a resource through a gene expression pattern in time. Various chains of such resource processing steps reflect different paths of nutrient conversions, such as occurring in the microbial nitrogen cycle. For example nitrogen can be cycled through few, large steps: , or with intermediate metabolites: .

In both the local and null model, the above described interaction between organism and environment is embedded on a two-dimensional grid (Figure 1A), where each grid site contains a single resource and at most one individual. Given a grid site without an individual, the organisms in the 8 neighboring sites compete for the opportunity to reproduce and place a daughter in the empty site. This competition is based on how well each individual can process the resource at the empty site: the longer an individual's bite, the larger is its probability to reproduce. Naturally, during reproduction mutations may occur (Figure 1C).

As a consequence, in the local model a lineage of fit individuals shapes the resource distribution in its vicinity. This causes an effect over several generations with respect to what resources are available in the local environment and thus impacts the evolution of the local population or community. We compare such local change to the null model in which we remove this opportunity of shaping the local environment. In the null model we randomly relocate all individuals on the grid at the end of each time step. This leaves the process of local competition and reproduction in place, but destroys any phylogenetic relationship between neighboring individuals – in effect individuals are not located close to their parent anymore – and removes the multi-generational effect of a lineage establishing itself in a patch and shaping its local surroundings (though a global effect remains).

### Model description

#### Grid

By default the grid is initialized with an identical resource at each site and a homogeneous population of size 8000 (individuals are randomly placed, at most one per grid site). Both the initial resource and individual have been randomly generated in advance and are reused in replicate runs. In addition to the argumentation in the Introduction on the use of space, the grid allows for a computationally efficient method of competition among individuals and is biologically sound, as organisms virtually always live in a spatial system with a certain degree of locality.

With a standard grid size of 100 × 100 and a fixed death rate per individual of 0.1, the population size is on average 9000 and approximately 1000 new individuals are born each time step. Note that in the null model, the individuals are mixed each time step, which means each individual is moved to a random location on the grid.

#### Resources

A resource is a bit string of length *n *(default *n *= 64) and can be altered into another resource by rotation from right to left. A minimal rotation of zero bits returns the original bit string, as does the maximal rotation of *n *bits. Thus the 'chemical universe' consists of *n *different bit strings, each a rotated version of another, with a total of *n*^2 ^= 4096 transitions or reactions between them. We identify each resource by the number of rotation steps performed on the original bit string. As a reference, the input bit string in Figure 1B is the original resource used in the main results. Furthermore, resources diffuse by Margolus diffusion [37], by default one diffusion step per time step.

#### Genotype and network

The genotype of an individual is a single chromosome, on which genes with their binding sites are placed. From this chromosome a Boolean threshold network is computed that can process resources. To this end each gene and binding site have a binding specificity: an integer number. The network is constructed by connecting genes with binding sites if their binding specificity is equal. Thus all genes with specificity 3 bind to all binding sites with a specificity 3, possibly connecting multiple source genes with various target genes. We allow for self-loops and parallel connections. There are three categories of genes: input genes (with a binding specificity in the range [0, 7]), processing genes (specificity in [8,14]) and output genes (binding specificity 15, which is for identification only, i.e. there are no binding sites with specificity 15). Not having any output genes is lethal, the other genes are all optional.

#### Resource processing by the network

Resource processing is modeled as gene expression dynamics: genes have to be activated and inhibited to process a resource. First, the first 8 bits of the input string are assigned as starting states to the input genes by binding specificity. The rest of the genes are set to zero. As an example: all genes 4 are assigned the state of the fourth bit of the bit string. Note that input genes may be deleted by mutations and the sensing of bits may thus be impaired. Second, after some calculation steps (default 2) the task of the network is to reproduce the bit string as a gene expression pattern through time on its output genes. The state of the output gene is read and matched to the bit string. If there are multiple copies of the output gene, the output is considered 'on' if at least one of the output genes is 'on', else it is 'off'. As soon as a mismatch is detected, the network updating is stopped. We refer to the resulting stretch of matching output bits as a "bite". Both the calculation steps and the expression output steps are done by updating the Boolean network in parallel [38,39]. For a gene this is defined as:



with  the expression state of gene *i *at time *t *(s = 0 is 'off' and 1 is 'on'), *w*_*ij *_the weight (1: activating and -1: inhibiting) of binding site *j*, and *θ*_*i *_the threshold of expression of gene *i *(*θ *∈ {-2, -1, 0, 1, 2}). The network dynamics are performed in one simulation time step and the number of updates may vary from 2 to *n *+ 2 depending on when a mismatch occurs.

#### Reproduction

If no individual is present at a site, the neighbors compete for the empty site in order to reproduce and place a daughter at the empty site. The neighborhood *nbh *consists of the 8 adjacent sites, also known as a Moore neighborhood. The competition consists of each neighbor trial processing the resource of the empty site as described previously. The fitness of an individual, *f*, is defined as



with *l *the length of bite, *g *the genome size (number of binding sites and genes), *pen *the genome size penalty coefficient, and *σ *the selection coefficient. From the fitness we calculate a relative fitness *r*_*i *_of each individual *i *in the neighborhood: *r*_*i *_= *f*_*i*_/∑_*j *∈ nbh _*f*_*j*_. Next, one is selected according to the fitness proportional selection scheme. Reproduction consists of duplicating the chromosome, applying mutations and dividing into two daughters. Subsequently one of the two daughters replaces the parent, the other is placed in the empty grid cell. And, the rotated resource (waste product) of the winner replaces the resource at the empty site.

#### Mutations

On the genes and binding sites mutational events have been defined as follows. Genes may (a) duplicate, which includes the accompanying binding sites, (b) be deleted, which also includes the binding sites of the gene, (c) have their expression threshold mutated to a random other threshold value, and (d) have their binding specificity changed to a random other value. Thus the effect of a change of binding specificity may be that a lost gene is rediscovered.

Binding sites may (a) duplicate, where a copy of a binding site is inserted in front of a random gene, (b) be deleted, (c) have their weight *w *changed from activating to inhibiting and vice versa, (d) have their binding specificity changed to a random other value. Default rates of gene and binding site mutations are mentioned in Figure 1.

### Analysis

#### Phenotype

For an individual we can calculate the bites it produces for each resource. This results in 64 bites, each in the range [0, 64). Next, we store the bite lengths in a vector, and we define this vector as the phenotype of an individual. A vector index corresponds to the number of rotation steps from the original resource. The phenotype is a measure of overall performance, as an individual is evaluated on only few resource in its lifetime.

#### Network dynamics

If networks are allowed to continue generating output bits after the first mismatch with the input resource, repeating patterns of zeros and/or ones are observed. In other words, if we view a network as a dynamical system, it initially moves through a transient and then settles in a fixed point or cyclic attractor. Per individual, we calculate the transients and attractors for all resources. Next we group individuals by these network dynamics as a measure of diversity in the population. In addition, such dynamics allow us to examine how the different bites are generated. Even if the phenotypes of individuals are equal, the network dynamics may be different.

#### Phylogeny tracing

Every individual has a unique identification tag and its parents' identification tag. We record these relationships, and in addition, we periodically log a sample of the population (every 1·10^4 ^time steps) and the entire population (every 2.5·10^4 ^time steps). This allows us to compute the 'true' ancestry of individuals in a phylogenetic tree, to correlate branching depth to phenotypes, and to display the evolution of various properties.

### Ecosystem

In order to visualize the ecosystem at a specific time point, we depict it as a network with resources as nodes and bites that transform one resource in another as edges. In the resulting figures the following ecological concepts are easily observed.

#### Shortest cycle

The rotations of bit string resources leads to cycles. As we have *n *= 64 resources, a cycle of bites has a maximum length of 64 (i.e. each bite is one rotation step and omitting self-overlapping cycles). It follows that shorter cycles have longer bites, which means higher fitness for the individuals involved. This brings us to the shortest cycle: the cycle that contains the smallest set of bites that cycles a set of resources, where each bite can be performed by at least 5 individuals at a time step.

#### Crossfeeding

A special case of division of labor is crossfeeding: the shortest cycle is formed by the cooperation of multiple lineages. To be more precise: we compute the shortest cycle and take a 10% sample from the population (≈ 900 individuals). Crossfeeding is present if the shortest cycle cannot be performed by a single group of phenotypically identical individuals, with the group having at least 5 individuals.

#### Ecological simulations

The stability of crossfeeding is studied with so-called ecological runs. Such runs are initialized with a population and accompanying environment – that is the resources on the grid – from a specific time point of an evolutionary run. In these runs we omit the mutational process and therefore eliminate much of the 'noise' generated by mutants and their aberrant bites.

## Results

Due to the nature of resource processing (rotating a bit string) the ecosystem evolved to contain one or more, overlapping, cycles in which the bites neatly map onto each other. This optimization of resource cycling was selected for indirectly, and importantly, implied a frustration in the evolutionary process. This frustration is a crucial feature of the dynamics of our model: in the long run it can pay off to not increase the direct fitness, but to produce resources which the offspring or local community can process well. We refer to this secondary level of selection for a cycle of bites as indirect selection.

Therefore in this study we do not focus on evolution as an optimization process of resource processing (i.e. maximizing bite lengths). Instead, the emphasis of our studies is on long-term indirect selection, its effect on resource dynamics, and how evolution structures the ecosystem with respect to the population and the individual. We investigate this by varying two parameters: the spatial setting and direct selection. We compare the outcome of the local and null model, and we take 3 levels of direct selection (*σ *= 0.2, 1.0 and 5.0). For each level we have performed 25 runs of both models (150 runs in total). Note that when direct selection is weaker, indirect selection can play a more important role. Also, drift plays a larger role in this case, but for the values of *σ *used (and 5 times smaller) the drift does not prevent adaptation. Thus, we will refer to more direct and less indirect selection (and vice versa) when we simply change the level of direct selection.

The results are organized as follows. We shortly introduce both models, followed by a detailed view of what kind of individuals initially evolve and what the initial ecosystem looks like. Next, we study population diversity and how it evolves over time: how does the ecosystem "solve the problem" of cycling resources? We focus on questions such as: do local interactions among individuals promote or inhibit cooperativity, such as crossfeeding? Do individuals evolve to cycle resources 'all by themselves'? How do individuals and their community structure change if we vary direct selection?

Additional simulations have been run to test for the robustness of our main body of results against various parameter changes, such as mutation rates, starting networks, different resources and resource size (Additional file 1: Text1).

### Overview of the local and null model

**Figure 2:**
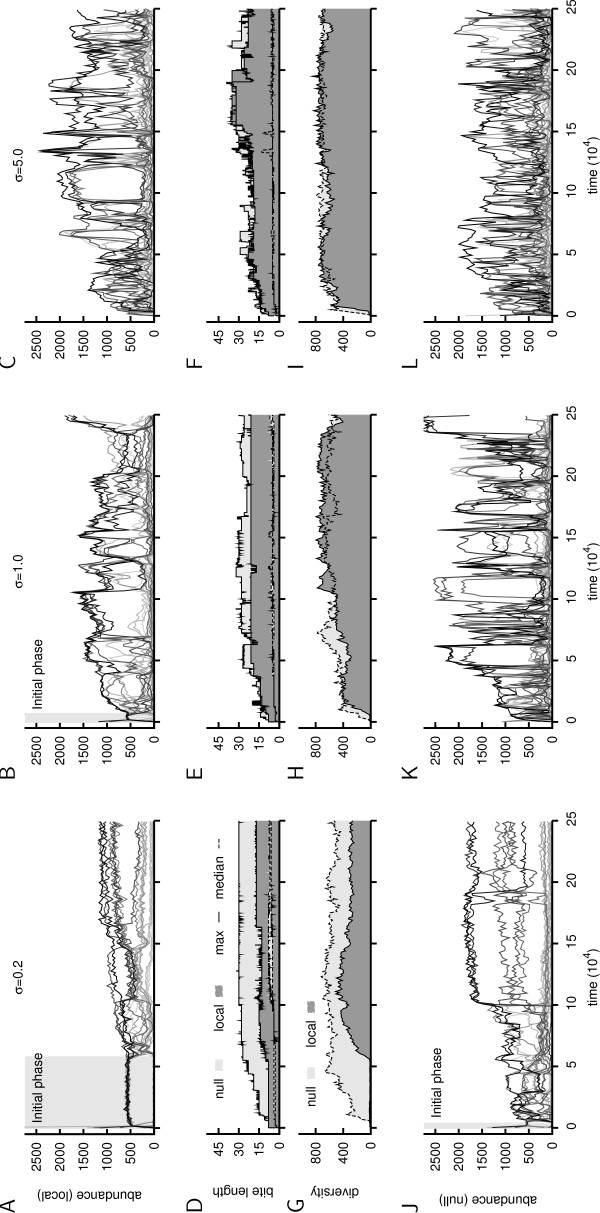
**Resource abundance, population fitness and population diversity**. A – C and J – L. The 20 most abundant resources are plotted through time. The darker a curve, the higher the abundance of this resource throughout the run. The top and bottom row contain, respectively, runs from the local and null model (for each selection regime a run). If visible at this scale, the initial phase is indicated by a gray background. D – F. Maximum and median fitness of the population through time. Dark shaded areas indicate the local model, light shaded areas the null model. G – I. Diversity in the population measured as the number of different network dynamics through time. Each 1000 time steps 1000 individuals were sampled and grouped by their network dynamics. The number of different groups is plotted. Grouping by phenotype gave qualitatively similar results.

In Figure 2 we show for both models and each selection regime a representative run. Clearly, both models show that from low to high selection (and thus from relatively high to low indirect selection) the resource dynamics in the environment become more turbulent. Importantly, there is also a clear difference between the models as the local model is more 'quiet', or stable in its resource dynamics. With respect to fitness (Figure 2D–F), we find that in both models the difference between maximum bite and median bite is large, and median bite length increases hardly. The most striking property of the diversity (Figure 2G–I) is that the sudden burst of diversity corresponds with the end of the so-called initial phase. It is also interesting to note that a diversity of ~600, given a sample size of 1000 individuals, implies that there is a lot of diversity in the populations at *σ *= 1.0 and 5.0, and in the null model at *σ *= 0.2. Finally, we highlight the dynamic nature of the system on the level of an individual's phenotype (and therefore also its genotype): turbulent resource dynamics and the gain and loss of maximum bite lengths indicate the many invasions of new lineages and the extinction of old ones.

**Figure 3:**
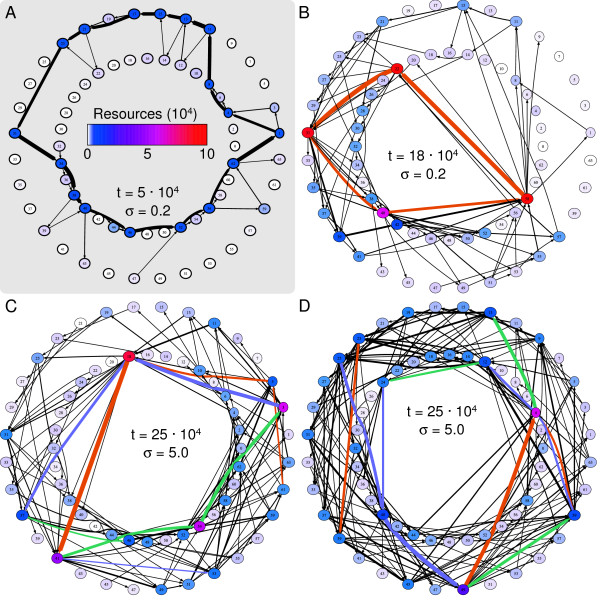
**Ecological views of several runs**. Each 'wheel' shows the 64 resources (nodes) and the bites (edges) that rotate one resource into another. For visualization purposes even and odd numbered resources are plot on the inner and outer circle, respectively. Resources are colored by abundance, see the legend in panel A. The edge-width is logarithmically scaled according to popularity: the more a bite occurred the thicker the edge. In B, C and D the shortest cycles are colored to distinguish up to 3 different phenotypic groups (orange, purple and green). This coloring does not indicate any relationship between the phenotypes in different 'wheels'. A and C are taken from runs with local feedback (Figure 2A and C), B and D are from runs of the null model (Figure 2J and L). A. Run with low direct selection (*σ *= 0.2) at time 5·10^4^, which is still in the initial phase of evolution. B. Run with selection *σ *= 0.2 at time 18·10^4^. The shortest cycle (22, 31, 40, 58) is performed by a single lineage. C. Local model run with *σ *= 5.0 at time 25·10^4^. The shortest cycle (3, 18, 41, 54) is composed of three different phenotypic lineages. D. Null model run with *σ *= 5.0 at time 25·10^4^. There several shortest cycles, composed of multiple lineages. One of these cycles is: 4, 12, 24, 40, 49.

From the runs in Figure 2A, C, J and 2L we have taken a single time point and visualized the ecological interactions between individuals and resources (Figure 3). Again we observe two 'gradients' from rather simple dynamics to complex interwoven cycles of resource modifications: both from local to null model, and from low to high direct selection. Moreover, under low direct selection, we find a single lineage performing the resource cycling, while for high selection multiple cooperating lineages are shown.

### The first resource cycles

We consider the example run with local feedback shown in Figure 2A. It can be divided into two periods: an initial phase characterized by an equal abundance of a subset of resources, and the long term evolution of the run in which resource numbers varied from equilibrium dynamics to turbulent patterns in time. The initial phase as we observed it in the example run was found across all runs, though its duration varied and was shortened with higher values of *σ *. We now present this period in terms of the sequences of zeros and ones that individuals output, allowing us to show the mechanics of our model. This nicely complements the higher-level ecosystem view we take in the subsequent study of long-term evolution.

In the initial phase the population was characterized by individuals that produced simple output bit strings in response to resources. We found that in the example run the networks generated first a transient of zeros, followed by the point attractor of value one. In Table 1 all bites are enumerated that lead to reproduction at time step 5·10^4^. Many bites were still small (2 or 3 bits) and thus of low fitness. All bites were of low complexity: for instance, both the most abundant (01) and most fit (01111111) would be generated by the same transient and attractor. Only the most abundant output would have a mismatch much earlier. Also, a single output 'strategy' was often applied to multiple resources (see third column in Table 1). To study which bit strings individuals could produce, we analyzed the underlying network dynamics. We found that the strings were generated by a single lineage with the following 5 typical outputs: 00001*, 0001*, 001*, 01* and 1*, where the star signals the continued emission of the last bit. Thus the individuals were capable of modulating the transient length depending on the input before ending in a point attractor. This behavior was typical for the initial phase of the runs.

**Table 1:** Output bit strings at time = 5·10^4 ^for the example run of Figure 2A.

output	count	bites
01.......	291	11→13, →13→15, 15→17, 21→23, 38→40
0011.....	208	4→8, 17→21, 34→38, 52→56
011......	132	31→34, 62→1
001......	94	1→4, 8→11
011111...	54	46→52
000011...	46	40→46
01111111.	44	23→31
000111...	41	56→62
		
11.......	24	6→8, 19→21, 32→34, 36→38, 44→46
00.......	21	1→3, 4→6, 17→19, 34→36, 52→54, 56→58
1........	12	3→4, 12→13, 22→23, 39→40
000......	8	40→43, 56→59
0........	6	11→12, 13→14, 21→22, 38→39, 46→47, 62→63
0000.....	5	40→44
111......	5	59→62
11111....	1	47→52

In the first column output bit strings are shown, with mismatched bits as dots (.). As the length of a correct output is the main determinant of an individual's fitness, one can easily differentiate between high and low fitness bites. The second column contains the number of individuals which generated the output. Matching resource rotations (bites) are given in the third column, corresponding to the ecological snapshot in Figure 3A. The upper half of the table contains all bites from the shortest cycle, the lower half contains 'mutant' bites.

**Figure 4:**
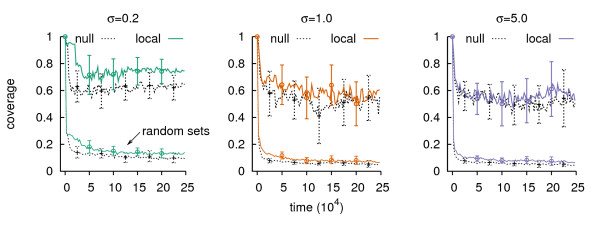
**Resource coverage of shortest cycles**. Mean coverage of the grid by resources on the shortest cycle, and random sets of resources. Coverage is expressed as a fraction of the total grid size. The random sets are composed of resources selected at random from the grid such that the size of the random set equals the number of resources on the shortest cycle.

In the ecosystem snapshot of time step 5·10^4 ^(Figure 3A) we observed a dominant cycle that was composed of popular bites and abundant resources. As expected, this was in concordance with Table 1: row 1 to 8 contain all bites from this shortest cycle. The positive correlation between shortest cycle and resource abundance was a general property that held in all runs. The resources on the shortest cycle occupied over half of the grid, while a randomized ensemble of resources covered 3 to 4 times less space (Figure 4). We can understand this as an equilibrium between the dominant lineage and the mutants that were present. The dominant lineage maintained resources on the shortest cycle, and possibly channeled other resources onto the shortest cycle. On the other hand the various mutants took bites such that the resulting resources were likely not on the shortest cycle anymore. This explained the equilibrium we observed in Figure 2A.

Furthermore, as the shortest cycle was a concatenation of relatively large bites (compared to the bite-composition of other cycles, which were by definition longer), the dominant lineage would often win the competition for reproduction. Thus while this lineage established itself in the population, it out competed the other individuals and there would be an increase in shortest-cycle resources, until an equilibrium was reached. The result was a shortest cycle that was composed of abundant resources and the bites were performed by the dominant lineage.

Summarizing, our ecosystem was initially composed of a population of simple individuals that shaped their environment with a low variety of processing steps. Resource cycles quickly emerged, of which the shortest cycle of bites is an important property of the system. In all runs, this first phase of evolution abruptly ended with the innovation of oscillatory output patterns (Additional file 1: Text1). It was accompanied by a sudden dramatic increase in phenotypic diversity (Figure 2D–F). And while the initial phase had been rather similar for all runs, the long term outcomes were different, as we show next.

### Evolutionary stagnation

From a detailed account on the initial phase, we now move to an ecosystem point of view. As mentioned previously, we focus on the effect of indirect selection. We do this by studying the differences in behavior of the local – and null model, and by examining the effect of different levels of direct selection. We show that relatively high indirect selection, especially by local feedback between individual and its environment, leads to evolutionary stagnation in three different ways.

#### Resource dynamics

**Figure 5:**
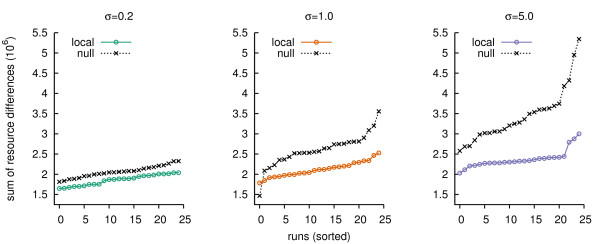
**Resource turbulence**. For each run, we calculate for all resources the sum of absolute changes in resource abundance through time, and then sum over the resources. Thus we arrive at a single number indicating how turbulent resource dynamics have been through a run. For each selection regime the difference between the local and null model is statistically significant. For *σ *= 0.2, 1.0 and 5.0 the Wilcoxon rank-sum test (alternative hypothesis: local less than null) results in, respectively, *p *< 2.73·10^-6^, *p *< 5.97·10^-8 ^and *p *< 1.38·10^-12^.

In the comparison between the local and null model, resource dynamics in runs with local feedback were less turbulent and more often in equilibrium (Figure 2 and Figure 5). In addition, the local model showed only a minor increase in turbulence from low to high *σ*, while for the null model this increase was much larger (Figure 5). Thus local feedback enhanced a stable cycling of the same resources (i.e. indirect selection for a cycle of resources), which implies fewer mutants established in the populations and therefore a slow down of the evolutionary process.

#### Bites

Next, we considered the bites that occurred in the population over time. As argued previously, in case an individual influences its local environment, there is an incentive to have resource modifications that nicely follow up on each other: if an organism can increase the probability of its descendants having an "easy bite", this will lead to more successful offspring in the long run. On the other hand, in the null model this process is not present as local kin relationship is destroyed by the mixing of individuals. How does this difference reflect in the distributions of bite lengths over time?

**Figure 6:**
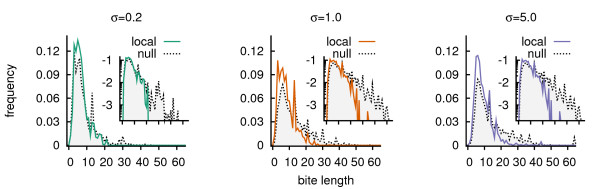
**Frequency distributions of bite lengths that lead to reproduction**. The mean distributions are shown, summed over the period t = 12.5·10^4 ^to 25·10^4^, and based on 25 runs of both the local and null model. The insets in the panels show the frequency plot in log scale, such that the differences in the tail are highlighted.

The average bite that lead to reproduction in all selection regimes was around 4–6, for both the local and null model. However, as we observed clearly for low direct selection (*σ *= 0.2), the local feedback resulted in less long bites (Figure 6, left panel). There were no bite lengths > 21, while the null model showed bite lengths up to 48. Also, for *σ *= 1.0 and 5.0 bite lengths in the interval [20,40] were less present in case of local feedback (Figure 6).

Considering that mutation rates were equal and genome lengths comparable, mutants arose at a similar rate in both models. Yet in case of local feedback mutants with larger bites failed to establish. The only difference between the two models was that in a spatial setting mutants were polluting their local surroundings and they were 'confronted' with this. That is to say, while the residing individuals were well-adapted to local resources and due to indirect selection also to the resources at the next time step, mutants found themselves among resources they could not process well. Thus most mutants could not establish a lineage locally and were out competed by the expanding lineages that cycled resources more efficiently. In the null model, however, by mixing the connection between individual and resource composition was much weaker. Mutants were not 'forced' to process their locally produced resources, hence it was easier for them to establish in the population. Thus, in the long run, mutants were subjected to stronger competition in the local model. And the higher indirect selection, the stronger this competition was.

From this also follows that for higher values of *σ *direct selection overruled indirect selection and mutants were able to invade. In concordance with this argumentation is the coverage of the grid by the resources of the shortest cycle. In Figure 4 we observed that while low selection had a high coverage (> 0.7), thus indicating few established mutants, *σ *= 1.0 and 5.0 showed a distinctly lower coverage. This points at alternative resource modifications taking place.

#### Shortest cycle

**Figure 7:**
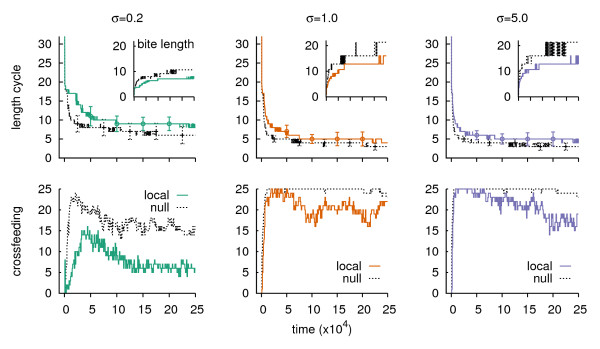
**Shortest cycles, their length and crossfeeding**. A. For the three selection regimes the mean shortest cycle with standard deviations is plotted through time. Inset panels show the corresponding average bite length on the shortest cycle. In all panels the mean and standard deviation are computed from 25 replicate runs. Using permutation tests, we established all three curves are significantly different (all *p *< 0.004). B. Number of runs with crossfeeding through time. In short, crossfeeding is present if the shortest cycle cannot be performed by a single group of phenotypically identical individuals, with the group having at least 5 individuals (see Methods).

As we have shown the core of the ecosystem dynamics was formed by the shortest cycle, we compared these cycles in the different settings. For *σ *= 0.2 not only bite length, but also cycle length had clearly stagnated (Figure 7A). Translated into bite lengths, a local feedback cycle of length 9 resulted in an average bite of length 7.11, while in the null model average bites were 64/6 = 10.7 bits long. Thus on average approximately three bits were processed less if local feedback was present.

Moreover, these differences in cycle length extended to *σ *= 1.0 and 5.0. At first sight a difference of one step may not seem significant, but as cycles become shorter the bites become progressively larger. For instance, for *σ *= 5.0, if local feedback was present cycle length was 5, which equaled a bite length of 12.8, while the null model had an average bite of 64/3 = 21.3. Thus there was a difference of more than 8 bits, which indicates quite some change in the network dynamics of the individuals.

Concluding, locality of the feedback between individual and environment enhanced the effect of indirect selection for cycling resources. Naturally, this was most obvious for low direct selection (*σ *= 0.2). However in comparison to the null model for all selection regimes we observed less change in the resource dynamics, a stagnated evolution towards long bites and a stagnated evolution of short cycles.

### Population structure

Given the evolutionary stagnation, we turn to the underlying population and community structure. We focus on long-term dynamics of the community for the different selection regimes in both the local and null model.

#### Crossfeeding

As described before, the innovation of oscillatory outputs marked a sudden increase in phenotypic diversity. From this large variety of different individuals cooperative cycling of resources emerged. Subsequently the lineages participating in shorter cycles, thus performing larger bites, took over and this was mirrored in a rapidly decreasing length of the shortest cycle (Figure 7A). Eventually a few groups of individuals became dependent on each other for resources on the shortest cycle. We labeled such structuring of the population as crossfeeding, and examined it in detail.

First of all, we looked at the evolutionary dynamics of crossfeeding. It is clear that for all three selection regimes the local model resulted in less crossfeeding than the null model. However, there was a stronger dependence on selection. As shown in Figure 7B, for low selection crossfeeding was a transient phenomenon. In both the local and null model, there was a peak before 5 ·10^4 ^and in ~55% of the runs a single lineage eventually took over, performing the shortest cycle by itself. Such was also the case in the example run we discussed previously, and its null model counterpart at *σ *= 0.2 (Figure 2A, J). The ecological network of the null model run is shown in Figure 3B. A single lineage performed the shortest cycle of length 4, while its close mutants created detours such as 31 → 39 → 42 → 58 and 58 → 11 → 15 → 21 → 31.

Contrastingly, runs with average and strong selection showed prolonged periods of crossfeeding (Figure 7B): it was the main long term evolutionary outcome. This impacted the length of the shortest cycle. Even though there was a 5-fold difference in *σ *between average and high selection, the lengths are comparable and substantially lower than for *σ *= 0.2 (Figure 7A). In Figure 3C and 3D snapshots show the partitioning of the resources among different phenotypes. Large bites were performed by specific phenotypes, while shorter ones were more prone to be shared (data not shown).

#### Ecological stability

Next, we studied the population dynamics of these crossfeeding runs. A population structuring while mutations occur does not imply ecological stability [23]. We performed ecological runs and tested if crossfeeding was maintained. The results stressed that for low selection the structuring is not stable: only few runs preserved crossfeeding (Table 2). However, for average and high selection all runs showed maintenance of crossfeeding. Thus in these cases we have a stable phenotypic partitioning of the population.

**Table 2:** Ecological stability of crossfeeding.

*σ*	runs	time (10^4^)		
		5	15	20
0.2	10	3/7	0/3	0/2
1.0	10	10/10	9/9	9/9
5.0	10	9/9	9/9	6/6

For each of the selection regimes, we have tested the ecological stability of crossfeeding in a random set of 10 runs, for populations taken from 3 time points. The total number of runs, including the ones that lack crossfeeding, is shown in the second column. The first time point is chosen early in the simulations, after the innovation event. The other two time points are indicative of the long term evolutionary dynamics. Each entry gives the fraction of runs with crossfeeding that show ecologically stability. A run is labeled as ecologically stable if crossfeeding is maintained in short ecological simulations (25 000 time steps, no mutations).

#### Phylogenetic basis

**Figure 8:**
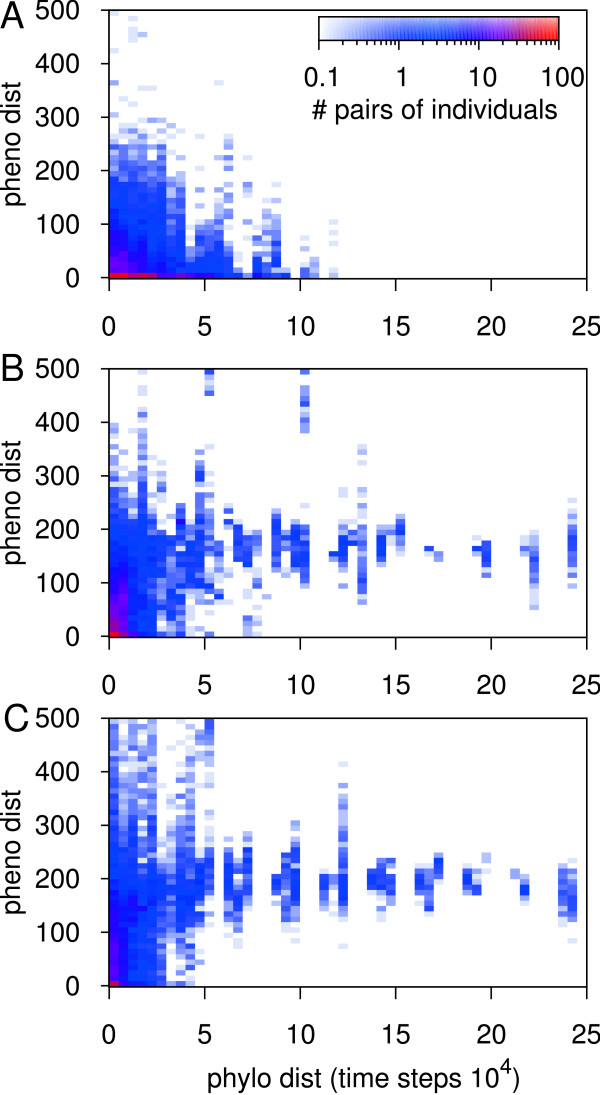
**Phylogenetic distance (phylo dist) against phenotypic distance (pheno dist)**. We computed phylogenetic trees of all runs with local feedback (for an example tree see additional file 2: Figure S1), and for each run we sampled a 1000 random pairs of individuals with a time of birth difference < 20 time steps and traced their last common ancestor. The phylogenetic distance is the difference in time of birth between the pair and their ancestor. Phenotypic distance is expressed as the Manhattan distance between two phenotypes. The colors, as given in the legend, give the number of pairs averaged over 25 runs. Note that the regular spacing in the data of B and C is an artifact of the periodicity of logging populations. Low, average and high selection are shown in A, B and C respectively (*σ *= 0.2, 1.0 and 5.0).

Still the question remained if multiple lineages were present if there was crossfeeding? We verified that the phenotypic structure of the population had indeed a phylogenetic basis, that is to say there was a genotypic grouping as well. In Figure 8 we show for the local model the phylogenetic distance between pairs of individuals against their phenotypic distance (see also additional file 2: Figure S1). It is important to realize that for short phylogenetic distances we observed mostly quasispecies variation among the phenotypes, while for large phylogenetic distances we have the actual population or community structuring.

One of the first observations was that all 3 figures have a red-colored peak close to the origin (0, 0), which implied that a large fraction of the pairs was both phenotypically and phylogenetically closely related. Second, low selection showed only limited phylogenetic diversity, which nicely corresponded to the evolutionary solution of a single dominant lineage. Third, there was an obvious difference between low selection and the other two regimes. While for *σ *= 0.2 the population remained similar as branch depth increases, for the other two average phenotypic distance increased. Furthermore, the latter two showed deep branches of dissimilar pairs of individuals. In fact, considering some pairs of individuals had a last common ancestor almost 25·10^4 ^time steps ago, in a few runs the population must have split into separate lineages only a relatively small period after the start.

Overall, the null model agreed well with the observations above. There was, however, one clear difference. We found that the last common ancestor was quickly different (Additional file 3: Figure S2), contrasting the rather long stretch of similar ancestors found in the local model. This indicates that lineages diverged, and thus specialized, quickly. Such faster divergence of the population is facilitated by mixing: the spread of a new adaptation is not limited by space, hence a mutant may establish faster.

In summary, both the local and null model show a clear dependency of crossfeeding on the strength of indirect selection, and this is most obvious along the different degrees of direct selection. High indirect selection – that is low direct selection or local feedback – often resulted in a single lineage performing a relatively long shortest cycle. With decreased indirect selection, via average and high direct selection or mixing of the population, prolonged periods of crossfeeding were observed. This crossfeeding was based on a phenotypic and phylogenetic differentiation in the population, and was ecologically stable.

For high direct selection (*σ *= 5.0), indirect selection was relatively low. Most of the decline in crossfeeding was not caused by indirect selection, but by an impressive result of the evolutionary process. In 4 out of 25 runs with local feedback and 1 null model run a lineage evolved that was capable of outputting a correct sequence of 64 bits for a specific resource, as one can also observe in Figure 6. This specialist lineage out competed everyone and thus dominated the population. Though impressive, we regard this result as an artifact at the boundary of interesting behavior in our model.

#### Individuals

From the population and community structure we concluded that strong indirect selection favored the evolution of generalist lineages. If we look at the individuals of the runs, an interesting question is to assess their 'smartness'. How well would they process all the resources by themselves? A straightforward definition of smartness is the sum of an individual's phenotype. Thus per individual we established for 64 resources its corresponding bite length and summed these lengths.

**Figure 9:**
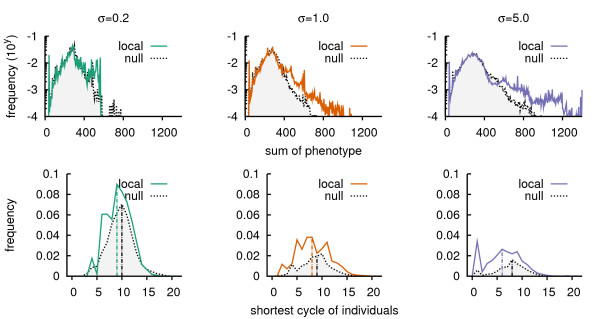
**Distributions of individual 'smartness'**. In both series of frequency plots we have taken per 1000 time steps a sample of 100 individuals, over the interval [12.5·10^4^, 25·10^4^]. With 6 × 25 runs this results in 6 data sets of 325000 individuals. A. Frequency plot of smartness defined as the sum of an individual's phenotype. Note the logarithmic y-axis. B. Frequency plot of smartness as an individual's shortest cycle. We calculated the shortest cycle given the presence of the most abundant resources at each sampling point. A minimal number of resources was selected such that the grid was covered by a 0.95 fraction. The three panels show the distribution of shortest cycle lengths of individuals that could actually perform a cycle. For individuals that were incapable of doing so see additional file 4: Figure S3.

For each setting (3 selection regimes, local and null model) we sampled 3.25·10^4 ^individuals in the second half of the runs, from 12.5·10^4 ^to 25·10^4^. In Figure 9A the resulting distributions of smartness are shown. In all cases there was a peak of 'stupid' individuals close to zero. These were simply mutants that had an extremely deleterious mutation. Secondly, all showed another peak around 300. Thus independent of the selection pressure or spatial setting most individuals were equally smart. Thirdly, it was in the right tail of the distributions that we found distinct behavior for each *σ *. With increasing *σ*, individuals became smarter in both models, and even more so in the local model.

However, the following alternative definition of smartness resulted (partly) in the opposite observation. Previously we argued that the evolutionary stagnation of resources and their shortest cycles was caused by the indirect selection for cycling resources. To study how this affected individuals we wondered what shortest cycles would be generated by the individuals themselves (that is without crossfeeding). Thus we introduced a second definition of smartness: to have a short cycle of resources. We allowed the individuals to use only resources that were abundant during their lifetime, and we found a clear difference between individuals along the two 'gradients' of indirect selection. More individuals from the local model were capable of performing a cycle, and they also had a shorter cycle than individuals from the null model (for each selection regime: Wilcoxon rank-sum test, alternative hypothesis: local less than null, *p *< 2.2·10^-16^), see also Figure 9B and additional file 4: Figure S3. Also, an obvious decline in the number of individuals that can perform a full cycle, was observed from low to high direct selection. Note that in the latter case the individuals that are capable of performing a cycle, actually do this in fewer steps for higher *σ *.

At first sight it was perhaps paradoxical that different definitions of smartness lead to contrasting results. Considering the first definition – the sum of bite lengths – the explanation is that under strong direct selection, individuals with large bites are strongly selected for and reproduce. We examined runs with local feedback by hand and in a subset of them (~25%) we found that the evolution for large bites for many resources had been very successful. Local feedback enhanced the capability to integrate information and as a result phenotypic smartness ensued [40]. Hence the tail of smartness in Figure 9A. With respect to the second definition, this smartness is simply a consequence of indirect selection: in both the local and null model resources had to be cycled. Thus strong indirect selection, either via locality or low direct selection, enabled the evolution of individuals that cycle resources.

In addition the following argumentation highlights the role of local interactions, as compared to the mixed case. We established that indirect selection for resource cycling via a local feedback resulted in a trend for single lineages performing the cycling across all three selection regimes (Figure 7B). Due to the locality of the interactions, crossfeeding was slightly obstructed, and individuals would (and could) rely on their own lineage (i.e. neighbors on a lattice were most likely closely related). Contrastingly, the mixing of individuals in the null model facilitated crossfeeding and due to the higher long-term evolutionary stability of crossfeeding (Figure 7B) individuals may have specialized.

## Discussion

In this study we examine the influence of an individual on its environment in an evolutionary setting. We let individuals evolve an abstract metabolic process of consuming resources and excreting waste products in a closed ecosystem. This introduced a new, indirect, selection for resource cycling. As a result a short cycle of resource modifications dominated the ecological dynamics, and depending on the strength of indirect selection eco-evolutionary phenomena such as stagnation, crossfeeding, generalists and specialists were observed.

### Evolutionary stagnation

In case of local feedback a lineage "co-evolves" with its surroundings, while mixing of the population (i.e. only global cycling) removes the relation between individuals and their local environment. We observe that local resource cycling results in stagnated evolution. Especially for low direct selection (*σ *= 0.2) evolution slows down drastically by the interaction between organism and environment. Looking at all selection regimes, we observe a lower throughput in the resource cycling (that is longer shortest cycles) compared to the well-mixed null model.

Previously, evolutionary stagnation has been observed in the context of predator-prey co-evolution on a lattice [24]. It was found that spatial patterns in the form of patches hindered the invasion of mutants. Recently, in a model on marine microplankton a different mechanism with similar outcome was found: an increasing number of sibling species competing for resources slowed down the evolution of the entire ensemble [41]. It is tantalizing to associate such evolutionary slowdowns with the well-known observation of morphological stability in many fossil species [42]. Could it be that interactions with the environment increase robustness of species on an ecosystem level? However, at the moment indirect, resource mediated ecological interactions among individuals have hardly been acknowledged as a potential mechanism that is contributing to the observed evolutionary stasis in the fossil record [41].

### Crossfeeding and self-sufficiency

In case of strong direct selection (*σ *= 1.0 and 5.0), crossfeeding lineages evolve and maintain with ease in both models. Two, occasionally three, genotypically and phenotypically distinct lineages form a cooperative community that is ecologically stable. The specialization on specific resources by different lineages could also be interpreted as a partitioning of the resources. Still, as the different lineages clearly depend on each others 'waste', crossfeeding is a more appropriate term for the ecological dynamics.

The common hypothesis is that crossfeeding in (microbial) populations originates from rate-yield trade-offs [1,5,7,43]. As we omit such thermodynamic constraints, an energy related trade-off is not present. However, due to the fact that a network has a maximum of 16 different genes, there is an 'information storage' trade-off. Nonetheless, we consider it unlikely that the maximum network capacity is reached, given the distribution tails in Figure 9A. Instead a more likely cause of generalization and specialization is the difficulty of evolving yet another recognition of a resource bit pattern or another long bite. In other words, the metabolism is practically not constrained as is the case for the rate-yield trade-off, but simply difficult [44].

If we look at the evolutionary trajectories of the various runs, an interesting succession of phases is found. In the initial phase there is a single lineage that performs the cycling: an individual-based ecosystem. The era of this lineage ends when oscillatory outputs are discovered. This innovation leads to a phase of cooperating lineages: a community-based ecosystem. The long-term behavior that follows depends strongly on the indirect selection for bites that neatly map onto each other. Local feedback and low direct selection both favor an individual-based ecosystem. In the long run the crossfeeding lineages tend to be replaced by a 'smart' generalist lineage that performs the cycling of resources by itself. On the other hand, both mixing the population and a high direct selection override the selection for resource cycling and foster a community-based solution of cooperating lineages. Thus the evolution of our models appears to show a gradient from smart populations with 'stupid', cooperating individuals to 'smart', self-sufficient individuals. Both the distribution of environmental knowledge over different lineages that compose a population, and the embedding of this knowledge in single individuals has been named "information integration" [45].

Such different modes of evolution have been reported previously in an evolutionary model on bacterial restriction-modification (RM) systems under pressure by a continuous stream of phages [46]. Either individuals were 'smart' in the sense that they contained many RM systems to guard against many phages, or the population was composed of mutually uninfectable bacteria with few RM systems. Similar results have been found in a study on individual-based versus ecosystem-based function approximation [45], where predators and scavengers subdivide a mathematical function in order to approximate it.

### Evolutionary innovation

We reported the innovation of oscillatory output patterns for both the local and null model, followed by the rapid takeover of the population by the lineage that 'discovered' the oscillations (see also additional file 1: Text1). Though it is tied to the modeling approach we apply, we find it extremely intriguing that a fitness gain may not be reducible to a single gene, but is found on a higher level, namely that of the network discovering a novel behavior. From a classical point of view one expects that as a mutant takes over the population, the phenotypic diversity decreases. In our model this is not the case, even if we decrease the mutation rate by two orders of magnitude. Instead, as the oscillatory behavior establishes in the population, there is a striking increase in phenotypic diversity, genome length and fitness (Additional file 5: Figure S4 and additional file 6: Figure S5). Our conjecture is that the innovation of a new mode of generating output makes a large variety of phenotypes accessible, and this is subsequently exploited by the evolutionary process. In other words, innovation leads to species radiation. Further research is necessary to establish the generality of this phenomenon.

### Modeling choice

From an ecosystem modeling perspective, we made choices that deserve some attention. First of all, we wondered if the fact that our ecosystem is closed with respect to the resources affects our results.

Therefore we performed various runs with a slightly modified system. Instead of rotating the resources, we let the individuals bite chunks from one side of the bit strings. Thus the bit strings become smaller and at some point are finished. A finished resource is then replenished with a new resource, identical to the original. As a result we now have an open ecosystem with an influx of nutrients. In this model we observed both stagnation and crossfeeding. Also the abundance of the different resources (leftovers of different lengths) changed over time in a qualitatively equal manner as in our default model. Thus our results are not directly dependent on the precise topology of the 'chemical universe'.

Second, questions regarding ecosystem evolution are often approached from an energy point of view, yet we have omitted any explicit constraints on the anabolic and catabolic activities of micro-organisms. This has allowed us to replace the thermodynamic constraints and complex nutrient pathways between bacteria, archaea and other micro-organisms with a much simpler chemical universe, and focus on the concept of having a feedback mechanism. As such our approach is a baseline study for the evolution of ecosystems.

Third, the local and null model differ on two features: the presence and absence of kin-relationships between neighbors, and the ability (or not) to create a specific local resource composition. Strictly speaking we cannot distinguish between these two in our current results. An alternative null model could be the mixing of the resources instead of individuals. Like in the current null model, the ability to create a specific resource composition for offspring would be eliminated, but neighbor relations would be preserved. We expect that whether or not individuals compete with their kin, also in this case indirect selection would be severely reduced.

Finally, ecosystems usually consist of (and are modeled as) food webs in which resources not only exist as 'edible' items in the environment, but also are immobilized in individuals and made available via predation and parasitism. In this study we have focused solely on individuals and their abiotic environment, and explicitly left out any direct interactions between individuals, except for competition. Thus in order to study the evolution of more complete ecosystems, we could in future work extend our model with the ability of individuals to evolve interactions among each other.

## Conclusion

The dynamics of our model amounts to how information on the environment is stored in the population and in single individuals. We used selection and the spatial setting to vary environmental structuring, population structuring and individual ecological roles. Most importantly we show the effect of different degrees of indirect selection on the eco-evolutionary solution via the contrast of local against global feedback and the different levels of direct selection.

In short, locality enhances the integration of information from the environment into single individuals. However, as a consequence resources are cycled more slowly and in that sense the ecosystem is less efficient. Though locality also plays a role in the evolution of crossfeeding, the latter is more dependent on the strength of direct selection. Crossfeeding is always observed after the initial phase of evolution, yet it is likely to be only a transient phenomenon if there is (relatively) weak direct selection. In contrast, strong direct selection leads to sustained crossfeeding, and therewith more efficient resource cycling and faster environmental change.

Despite our simplified *in silico *approach, it is suggestive to associate our results with the diverse range of ecosystems formed by microbial communities: from the individual-based, single-species ecosystem in a South African mine [47], simple endolithic ones [48] to complex soil communities [49].

## Authors' contributions

AC conceived of the study, analyzed the data and drafted the manuscript. PH participated in the design of the study, analyzed the data and helped to draft the manuscript. All authors read and approved the final manuscript.

## Supplementary Material

Additional file 1 **Oscillatory output and parameter dependencies**. We provide extra information on the nature of the oscillatory outputs generated by individuals. In addition, to assess the dependencies of our results on the model parameters, we varied several key parameters such as the mutation rates, starting networks and bit string length. Click here for file

Additional file 2 **Phylogenetic tree with leafs colored by genome size**. We plot the phylogenetic tree of a run with local feedback and *σ *= 1.0. Each 2.5·10^4 ^time steps a population is logged to disk and used in combination with ancestor tracing (see Methods) to build the tree. Nodes are individuals from the logged populations and ancestors at lineage-splitting events. In other words, we prune the tree for intermediate ancestors. The edges thus represent branches from last common ancestors, and are scaled and colored by time interval. For the coloring of the leaves the genome length, genes plus binding sites, is mapped to a color from yellow to red. The arrow in the top-left corner points to the ancestor in the initial population (triangle node). We observe an overall modest genome size, with occasional branches evolving toward long genomes. See also additional file 6: Figure S5. Click here for file

Additional file 3 **Phylogenetic distance (phylo dist) against phenotypic distance (pheno dist) in the null model**. We computed phylogenetic trees of all null model runs with *σ *= 1.0 and 5.0 (due to technical reasons data for *σ *= 0.2 was not available). For each run we sampled a 1000 random pairs of individuals with a time of birth difference < 20 time steps and traced their last common ancestor. The phylogenetic distance is the difference in time of birth between the pair and their ancestor. Phenotypic distance is expressed as the Manhattan distance between two phenotypes. The colors, as given in the legend, give the number of pairs averaged over 25 runs. Note that the regular spacing in the data is an artifact of the periodicity of logging populations. Average and high selection are shown in A, B respectively (*σ *= 1.0 and 5.0). Click here for file

Additional file 4 **Fraction of individuals incapable of cycling resources by themselves**. We have taken per 1000 time steps a sample of 100 individuals, over the interval [12.5·10^4^, 25·10^4^]. With 6 × 25 runs this results in 6 data sets of 325000 individuals. For each selection coefficient we find that local feedback (colored bars) results in a smaller fraction of individuals that cannot cycle resources on their own, compared to global feedback (light gray bars). This plot complements Figure 9B. Click here for file

Additional file 5 **Sudden increase in diversity**. The number of unique bites is shown for the first 10·10^4 ^time steps. The number of unique bites in an ecosystem is calculated by counting the number of different bites that occur each time step. This measure is directly related to the phenotypic diversity. If there is more variety in the ways individuals process resources, the number of unique bites increases. In the same manner a phenotypically uniform population will have a low number of unique bites, as one can observe in the initial phase of evolution (clearly visible for *σ *= 0.2). Furthermore, though we plot only the runs of the local model, the null model simulations result in qualitatively equivalent plots. Click here for file

Additional file 6 **Genome length through time**. Genome length through time for a run with local feedback and *σ *= 1.0. Genome length is defined as the sum of genes and binding sites. The total spread of different genome lengths at each time step is given by the gray dots, with the median and the 1st and 3rd quartile given by the solid and dashed black lines. We observe that the genome length penalty *pen *does not inhibit the evolution of large genomes. See also additional file 2: Figure S1 for the phylogenetic tree of this run. Click here for file
