# Supplementary material for: Evolution of resource cycling in ecosystems and individuals
Source: BMC Evol Biol. 2009 Jun 1;9:122. doi: 10.1186/1471-2148-9-122 (PMC2698886; doi:10.1186/1471-2148-9-122)
Supplement: Additional file 1 — Oscillatory output and parameter dependencies. We provide extra information on the nature of the oscillatory outputs generated by individuals. In addition, to assess the dependencies of our results on the model parameters, we varied several key parameters such as the mutation rates, starting networks and bit string length. [file 1471-2148-9-122-S1.pdf]

1 Supporting information

Oscillatory output

As explained in the Results, the initial phase of evolution consisted of individuals that produced simple output bit sequences. Such a sequence consisted of an initial transient of zeros (or ones) that switched to a perpetual stream of ones (or zeros) as the gene network ended in an attractor state.

In each simulation this initial phase ended as individuals evolved networks that ended not in a fixed point attractor, but in a cyclic attractor. The individuals apply these cyclic attractors to oscillate the sequence of bits at the output gene(s). We found simple oscillatory patterns at first, for instance `01*` or `110*`, with the star signaling the continued repetition of the pattern. As simulations progressed, evolution produced individuals that are capable of oscillating differently depending on the input patterns they observe, while the output patterns became more intricate: as an example `01101011110*`.

Such oscillatory output patterns enabled the individuals to process much larger parts of the resource bit string and hence gain a high fitness. Moreover, the oscillatory behavior of the network seems not reducible to a single gene, but is found on a higher level, namely that of the network topology. As described in the Results and Discussion, the discovery of this new class of behavior lead to a rapid radiation of new phenotypes in the population.

Parameter Dependencies

In order to assess the dependencies of our results on the model parameters, we varied several key parameters such as the mutation rates, resource diffusion rate and bit string length.

Mutation rates

We know from previous work that the network modeling formalism we apply has a large fraction of neutral mutations in its mutational neighborhood [Crombach and Hogeweg, 2008]. Therefore we have chosen the default mutation rates relatively high, as this increases the probability beneficial mutations are discovered. Hence we focus here on the effect of mutation rates that are lower than the default rates.

We ran simulations with mutation rates one order of magnitude smaller (e.g. gene insertion now is $16 \cdot 10^{-5}$). Except for slowing the speed of evolution, for instance under weak selection the innovation of oscillatory output may take almost $20 \cdot 10^4$ time steps (of a total $25 \cdot 10^4$ steps), we do not observe qualitatively different outcomes. An initial period is visible, followed by the invention of oscillatory output patterns. Also, as expected, only a small period of crossfeeding is found for weak selection, while sustained resource interdependence is observed for average and strong selection.

Different bit strings as resource

Our main body of results is based on a default resource. The resource was randomly generated and we do not expect any specific effects from our particular choice of bit string. Nevertheless we checked the validity of our results against ten other (randomly generated) bit strings of length 64. We observed qualitatively equivalent dynamics for resource dynamics, bite lengths, network diversity, crossfeeding and shortest cycle.

In addition we performed simulations ($\sigma = 1.0$) with a shorter bit string (length 32), and a twice as long bit string (length 128). For short bit strings, in 2 out of 3 runs there evolved a specialist that performed a 32 bit output and thus out competed the rest and dominated the population. In case of long bit strings, we performed 3 runs and observed rather quiet resource dynamics (though within the range of $\sigma = 1.0$), long shortest cycles ($> 10 \approx > 5$ for 64 bits) and extensive crossfeeding. The length of the shortest cycles is most likely affected by to the fact that a 128 bits generate many more input patterns to classify and process. The ‘problem’ of cycling resources became more difficult.

In case of short bit strings the environment is relatively simple and a single individual can specialize on a few resources, thus giving rise to extreme specialists that output 32 bits. With longer bit strings there is more information to be stored and input patterns to be recognized, resulting in extensive crossfeeding.

Different starting networks

Initially each individual has an identical genome and network. We replaced the default starting network with five different, randomly generated, networks, and for each we ran 3 simulations at $\sigma = 1.0$. We observe qualitatively equivalent dynamics as described in the main text.

Next, we ran 10 simulations with a smaller network (8 input genes, no processing genes, 1 output gene) at average selection ($\sigma = 1.0$). The resource dynamics were often rather ‘quiet’, yet crossfeeding was in 8 of 10 runs the evolutionary stable outcome. In addition, the distribution of bite lengths that lead to reproduction appeared to be an intermediate between low and average selection. Thus by making the network smaller, evolving large bites became more difficult.

References

A Crombach and P Hogeweg. Evolution of evolvability in gene regulatory networks. *PLoS Comput Biol*, 4(7):e1000112, 07 2008.
