# Supplementary material for: Evolution of resource cycling in ecosystems and individuals
Source: BMC Evol Biol. 2009 Jun 1;9:122. doi: 10.1186/1471-2148-9-122 (PMC2698886; doi:10.1186/1471-2148-9-122)
Supplement: Additional file 2 — Phylogenetic tree with leafs colored by genome size. We plot the phylogenetic tree of a run with local feedback and σ = 1.0. Each 2.5·104 time steps a population is logged to disk and used in combination with ancestor tracing (see Methods) to build the tree. Nodes are individuals from the logged populations and ancestors at lineage-splitting events. In other words, we prune the tree for intermediate ancestors. The edges thus represent branches from last common ancestors, and are scaled and colored by time interval. For the coloring of the leaves the genome length, genes plus binding sites, is mapped to a color from yellow to red. The arrow in the top-left corner points to the ancestor in the initial population (triangle node). We observe an overall modest genome size, with occasional branches evolving toward long genomes. See also additional file 6: Figure S5. [file 1471-2148-9-122-S2.pdf]

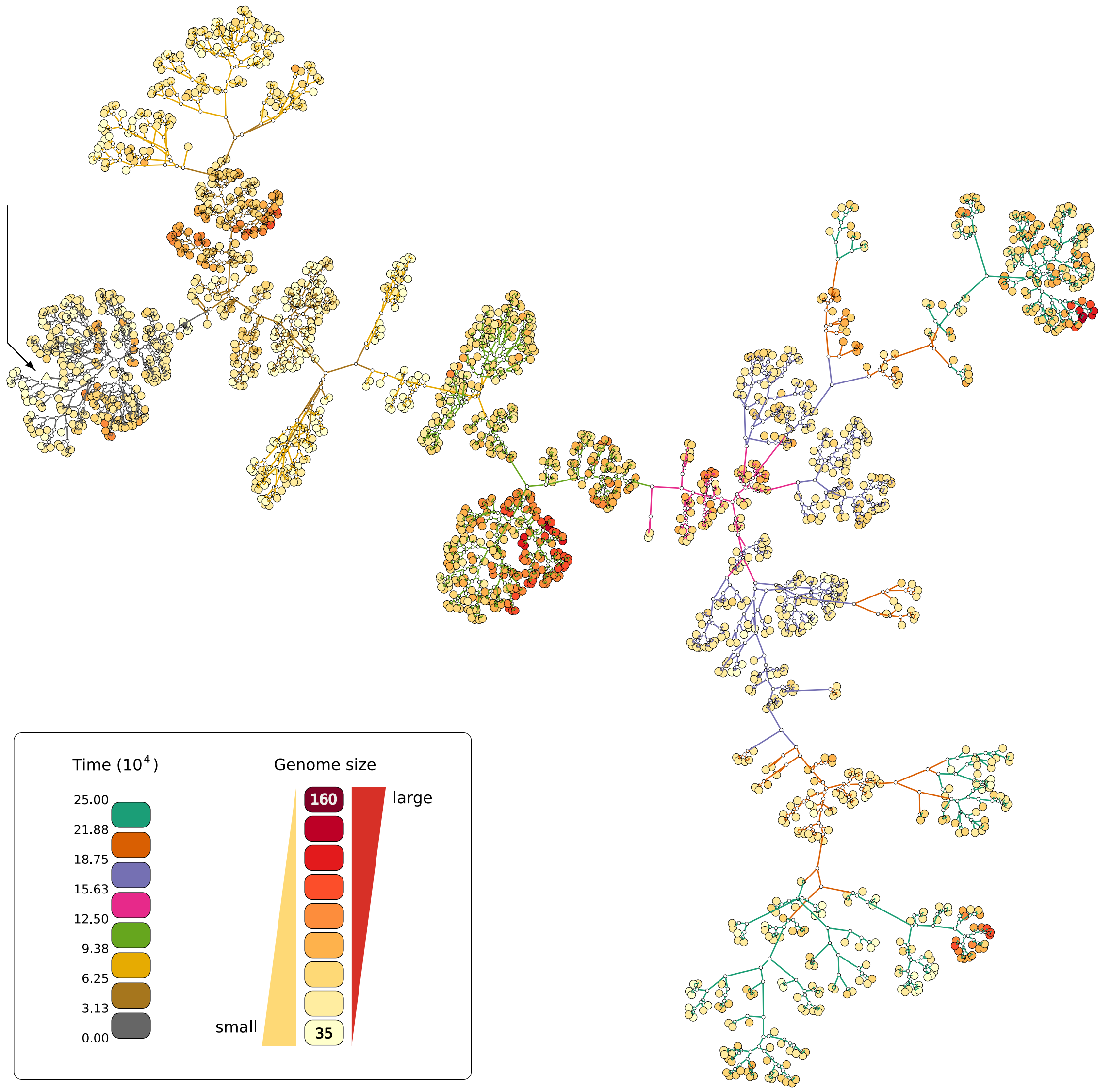

Figure S1: Phylogenetic tree with leafs colored by genome size. We plot the phylogenetic tree of a run with local feedback and $\sigma = 1.0$. Each $2.5 \cdot 10^4$ time steps a population is logged to disk and used in combination with ancestor tracing (see Methods) to build the tree. Nodes are individuals from the logged populations and ancestors at lineage-splitting events. In other words, we prune the tree for intermediate ancestors. The edges thus represent branches from last common ancestors, and are scaled and colored by time interval. For the coloring of the leaves the genome length, genes plus binding sites, is mapped to a color from yellow to red. The arrow in the top-left corner points to the ancestor in the initial population (triangle node). We observe an overall modest genome size, with occasional branches evolving toward long genomes. See also additional file 6: FigureS5.
