# Supplementary material for: Evolution of resource cycling in ecosystems and individuals
Source: BMC Evol Biol. 2009 Jun 1;9:122. doi: 10.1186/1471-2148-9-122 (PMC2698886; doi:10.1186/1471-2148-9-122)
Supplement: Additional file 3 — Phylogenetic distance (phylo dist) against phenotypic distance (pheno dist) in the null model. We computed phylogenetic trees of all null model runs with σ = 1.0 and 5.0 (due to technical reasons data for σ = 0.2 was not available). For each run we sampled a 1000 random pairs of individuals with a time of birth difference < 20 time steps and traced their last common ancestor. The phylogenetic distance is the difference in time of birth between the pair and their ancestor. Phenotypic distance is expressed as the Manhattan distance between two phenotypes. The colors, as given in the legend, give the number of pairs averaged over 25 runs. Note that the regular spacing in the data is an artifact of the periodicity of logging populations. Average and high selection are shown in A, B respectively (σ = 1.0 and 5.0). [file 1471-2148-9-122-S3.pdf]

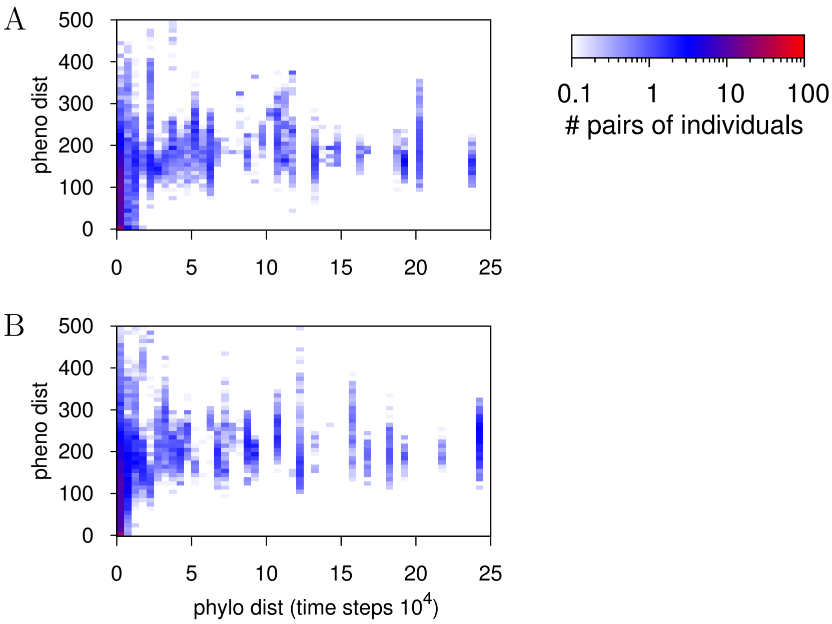

Figure S2: Phylogenetic distance (*phylo dist*) against phenotypic distance (*pheno dist*) in the null model. We computed phylogenetic trees of all null model runs with $\sigma = 1.0$ and 5.0 (due to technical reasons data for $\sigma = 0.2$ was not available). For each run we sampled a 1000 random pairs of individuals with a time of birth difference < 20 time steps and traced their last common ancestor. The phylogenetic distance is the difference in time of birth between the pair and their ancestor. Phenotypic distance is expressed as the Manhattan distance between two phenotypes. The colors, as given in the legend, give the number of pairs averaged over 25 runs. Note that the regular spacing in the data is an artifact of the periodicity of logging populations. Average and high selection are shown in A, B respectively ($\sigma = 1.0$ and 5.0).
