# Supplementary material for: Evolution of resource cycling in ecosystems and individuals
Source: BMC Evol Biol. 2009 Jun 1;9:122. doi: 10.1186/1471-2148-9-122 (PMC2698886; doi:10.1186/1471-2148-9-122)
Supplement: Additional file 4 — Fraction of individuals incapable of cycling resources by themselves. We have taken per 1000 time steps a sample of 100 individuals, over the interval [12.5·104, 25·104]. With 6 × 25 runs this results in 6 data sets of 325000 individuals. For each selection coefficient we find that local feedback (colored bars) results in a smaller fraction of individuals that cannot cycle resources on their own, compared to global feedback (light gray bars). This plot complements Figure 9B. [file 1471-2148-9-122-S4.pdf]

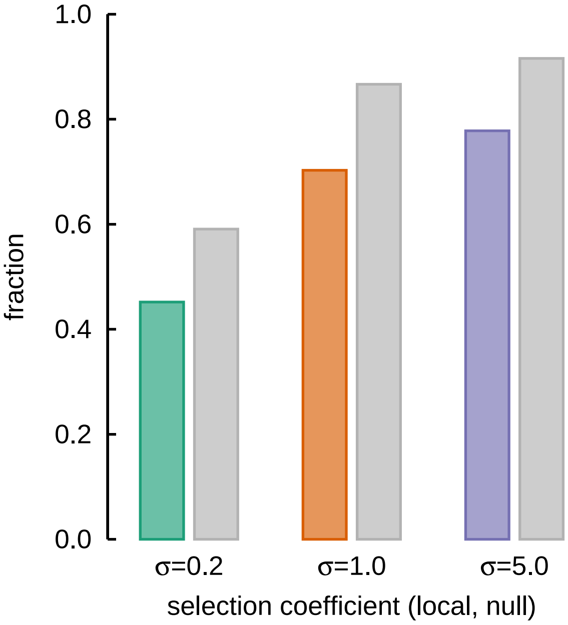

Figure S3: Fraction of individuals incapable of cycling resources by themselves. We have taken per 1000 time steps a sample of 100 individuals, over the interval $[12.5 \cdot 10^4, 25 \cdot 10^4]$. With 6×25 runs this results in 6 data sets of 325000 individuals. For each selection coefficient we find that local feedback (colored bars) results in a smaller fraction of individuals that cannot cycle resources on their own, compared to global feedback (light gray bars). This plot complements Figure 9B.
