# Supplementary material for: Evolution of resource cycling in ecosystems and individuals
Source: BMC Evol Biol. 2009 Jun 1;9:122. doi: 10.1186/1471-2148-9-122 (PMC2698886; doi:10.1186/1471-2148-9-122)
Supplement: Additional file 5 — Sudden increase in diversity. The number of unique bites is shown for the first 10·104 time steps. The number of unique bites in an ecosystem is calculated by counting the number of different bites that occur each time step. This measure is directly related to the phenotypic diversity. If there is more variety in the ways individuals process resources, the number of unique bites increases. In the same manner a phenotypically uniform population will have a low number of unique bites, as one can observe in the initial phase of evolution (clearly visible for σ = 0.2). Furthermore, though we plot only the runs of the local model, the null model simulations result in qualitatively equivalent plots. [file 1471-2148-9-122-S5.pdf]

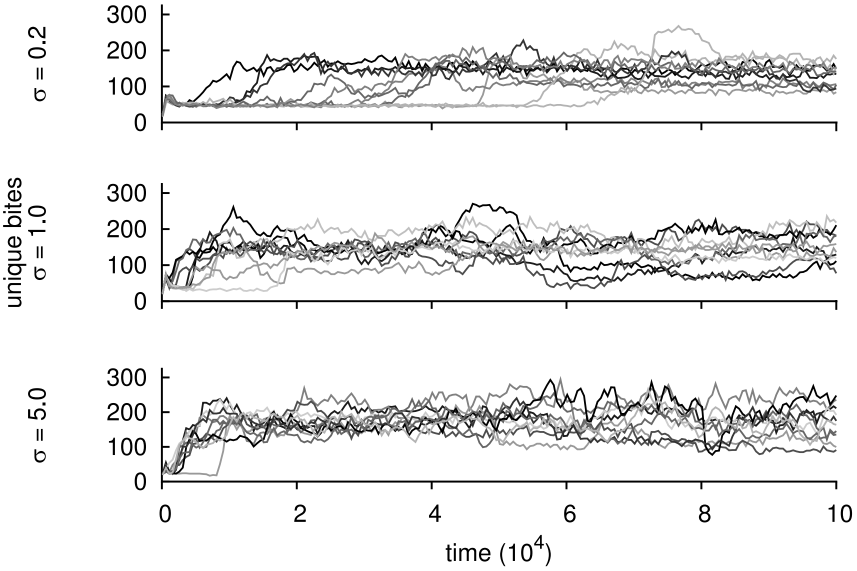

Figure S4: Sudden increase in diversity. The number of unique bites is shown for the first $10 \cdot 10^4$ time steps. The number of unique bites in an ecosystem is calculated by counting the number of different bites that occur each time step. This measure is directly related to the phenotypic diversity. If there is more variety in the ways individuals process resources, the number of unique bites increases. In the same manner a phenotypically uniform population will have a low number of unique bites, as one can observe in the initial phase of evolution (clearly visible for $\sigma = 0.2$). Furthermore, though we plot only the runs of the local model, the null model simulations result in qualitatively equivalent plots.
