# Supplementary material for: Evolution of resource cycling in ecosystems and individuals
Source: BMC Evol Biol. 2009 Jun 1;9:122. doi: 10.1186/1471-2148-9-122 (PMC2698886; doi:10.1186/1471-2148-9-122)
Supplement: Additional file 6 — Genome length through time. Genome length through time for a run with local feedback and σ = 1.0. Genome length is defined as the sum of genes and binding sites. The total spread of different genome lengths at each time step is given by the gray dots, with the median and the 1st and 3rd quartile given by the solid and dashed black lines. We observe that the genome length penalty pen does not inhibit the evolution of large genomes. See also additional file 2: Figure S1 for the phylogenetic tree of this run. [file 1471-2148-9-122-S6.pdf]

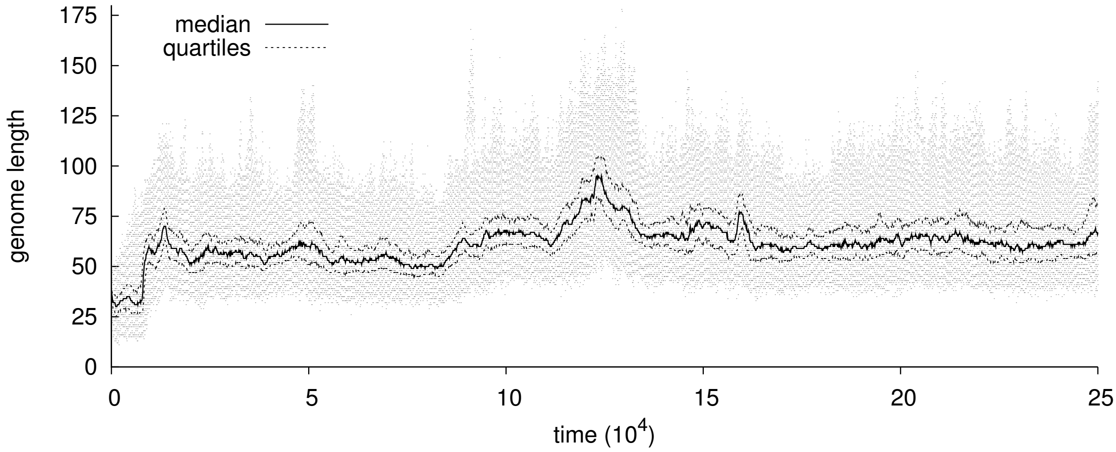

Figure S5: Genome length through time for a run with local feedback and $\sigma = 1.0$. Genome length is defined as the sum of genes and binding sites. The total spread of different genome lengths at each time step is given by the gray dots, with the median and the 1st and 3rd quartile given by the solid and dashed black lines. We observe that the genome length penalty *pen* does not inhibit the evolution of large genomes. See also additional file 2: FigureS1 for the phylogenetic tree of this run.
